# Experimental Study of Phlebitis Ointment Administration in Acute Superficial Thrombophlebitis

**DOI:** 10.1155/2018/2983195

**Published:** 2018-04-24

**Authors:** Guangzong Li, Gerhard Litscher, He Pang, Baozhong Yang, Daniela Litscher, Lu Wang

**Affiliations:** ^1^Department of Angiology (4th Surgical Department), Dongfang Hospital, Beijing University of Chinese Medicine, No. 6, 1st Section, Fangxingyuan, Fangzhuang, Beijing 100078, China; ^2^Research Unit of Biomedical Engineering in Anesthesia and Intensive Care Medicine and Research Unit for Complementary and Integrative Laser Medicine, TCM Research Center Graz, Medical University of Graz, 8036 Graz, Austria

## Abstract

Acute superficial thrombophlebitis is a venous system disease. Animal models with mannitol induced phlebitis were treated with an orally administered “phlebitis ointment.” 24 rabbits were randomly divided into 4 groups. The therapy group was treated with “phlebitis ointment” and a control group received “Mai Luo Shu Tong granules.” Levels of blood TNF-*α*, IL-6, CRP, and IL-1*β* were measured. The tissue expression levels of NF-КBp65 and PKC genes were evaluated. The therapy group showed a better improvement of the clinical status and similar vascular morphology than the control group. A blank group showed no vascular changes through pathological investigation. In contrast, significant vascular changes were seen in the model group. The control group showed slight vascular modifications. Small thrombi could be found in the lumen despite the intact tunica intima. Both control and therapy group showed less inflammatory cells infiltration than the model group and upregulation of NF-КBp65 and PKC genes. The phlebitis ointment reduced the levels of necrosis factor-*α*, interleukin-6, C-reactive protein, and interleukin-1ß. The expressions of NF-КBp65 and PKC genes, which are the primary mechanisms underlying the development of thrombophlebitis, were improved significantly in tissues of both therapy group and control group.

## 1. Introduction

Chinese herbs are recognized as effective treatment for vascular disease. However, most of the mechanisms of the therapeutic method are not clear. Acute superficial thrombophlebitis is a peripheral venous system disease. It is one of the common clinical and frequently occurring diseases. It is often secondary to superficial varicose, which occurs in the limb superficial vein of acute thrombosis. Clinical manifestations of shallow veins appear with indurations. The skin appears red and feels burning. Inflammation is locally associated with pain and tenderness. Some of the patients may be associated with skin fever, pigmentation, swelling, and other inflammatory reactions. Also the symptoms will be associated with fever, headache, and other systemic symptoms, which will seriously affect the work and life [1, 2].

Acute superficial thrombophlebitis has always been considered as a benign, self-limiting disease. It is not causing enough attention. This disease is often secondary to the lower extremity superficial varicose veins. About its causes, Virchow has proposed “three factors” for abnormal blood vessel walls, altered blood components, and abnormal blood flow [3, 4]. Modern clinical medicine often uses thrombolysis, antiplatelet aggregation, and anti-infection treatments. Long-term use of these drugs can cause bleeding and other side effects [5]. Chinese medicine treatment of thrombotic superficial phlebitis could have a positive effect. The phlebitis ointment used in this study is the internal preparation made by the Dongfang Hospital in Beijing. With its special characters of being easy to carry and easy to operate, this phlebitis ointment has also beneficial characters such as controllability of quality, long acting time, less side effects, and high compliance with patients. This ointment has been used in the above-mentioned hospital since 2015. The experiments put forward the scientific hypothesis of “oral using ‘Yiqi Huoxue jiedu' Chinese herbs to regulate proinflammatory cytokines-enhance cell biological activity in order to treat acute thrombotic phlebitis.”

Within this study we used a randomized controlled trial to observe the effect of phlebitis ointment on mannitol [6] induced phlebitis in rabbit ear vein. To study the application of “phlebitis ointment decoction” to intervene in the process of acute thrombotic phlebitis, we took the oral Chinese medicine preparation “phlebitis ointment” as the breakthrough point with our intention to investigate the pathological changes of blood vessels, inflammatory factors control (TNF- alpha, IL-6, CRP, and IL-1 beta), and changes of cell biological activity of signal transduction protein kinase PKC and NF-КBp65 [7, 8]. One goal of this study is to explore the related mechanism of acute thrombotic phlebitis.

## 2. Materials and Methods

### 2.1. Materials

#### 2.1.1. Experimental Animals

A total of 24 male and female healthy Japanese white rabbits were selected as experimental animal models (SCXK, 2014-0012, China). Of course, using the rabbit ear as a model does not fully reflect the situation in human patients. Nevertheless, it makes sense to use this model. The veins are relatively close to the surface and are easy to operate and to observe. Each selected rabbit, weighing between 2.5 kg and 3.5 kg, was housed individually and was given a week to adapt to the controlled environment. The light-controlled environment (12-hour light-dark cycle) was maintained at a temperature of 20 ± 1°C and a humidity of 50 ± 10%. The rabbits were allowed free access to food and sterile tap water except in the period of infusion with the test solutions. White rabbits, which displayed normal daily activities and revealed no systemic diseases, were chosen to proceed with further experiments. All the following experimental procedures were in accordance with guidelines issued by the National Institutes of Health. The procedure was approved by the Institutional Animal Care Committee of China Academy of Chinese Medicine Science.

#### 2.1.2. Drugs

Mannitol is a cell-impermeant, nonmetabolizable sugar administered intravenously as a hypertonic solution for acute and subacute reduction in brain edema. It is widely used in the clinical setting. Phlebitis is known to be a common side effect, which may result from apoptosis among vascular endothelial cells induced by mannitol and mediated by inflammatory mediators and mitogen-activated protein kinases. Clinical concentrations of mannitol activate tyrosine and stress kinases and induce apoptosis in endothelial cells [9]. However, mannitol can induce apoptosis. Clinical concentrations of mannitol induce apoptosis in endothelial cells, thereby damaging the vascular endothelium [10]. Mannitol may also activate inflammatory mediators and certain types of cells [10].

In the experiment, mannitol can make inflammation model increasing apoptosis in different endothelial cell of vascular system [11, 12]. Mannitol induced vascular impairment in rabbit vein endothelium. As a result, it produced phlebitis [13]. 20% mannitol solution has a higher concentration. As a result, it can cause obvious phlebitis changes, such as loss of venous endothelial cells, inflammatory cell infiltration, and perivascular edema [14].

20% (w/v) mannitol H11020861 was provided by China Resources Double-Crane Pharmaceutical Co. (Beijing, China). The acute superficial thrombophlebitis model of rabbit caused by 20% (w/v) mannitol has been reported elsewhere [15–18]. 20% (w/v) mannitol was infused into the veins of both ears of a rabbit. Each infusion was fixed at the puncture site, and all animals were used simultaneously in two ears.

Phlebitis ointment was prepared by Pharmacy Department of Dongfang Hospital, which is an affiliated hospital of Beijing University of Chinese Medicine (Beijing, China). The main herbal ingredients included 30 g of Shenghuangqi* (Astragalus mongholicus)*, 15 g of Danggui* (Angelica sinensis)*, 12 g of Chuanxiong* (Ligusticum chuanxiong)*, 20 g of Danshen* (Salvia miltiorrhiza)*, 6 g of Sanqi* (Panax notoginseng)*, 15 g of Chishao* (Paeonia veitchii)*, 20 g of Huzhang* (Polygoni Cuspidati)*, 9 g of Dilong* (Pheretima aspergillum)*, 15 g of Cangzhu* (Atractylodes lancea)*, 15 g of Huangbo* (Phellodendron chinense)*, 40 g of Jinyinhua* (Lonicera japonica)*, and 12 g of Shengzhizi* (Gardenia jasminoides)*. Referring to the standard dosage conversion guidelines were published by Professor Wei et al. in* Experimental Methodology of Pharmacology* [19]. The animal models were administered twice daily with a dose of 2.34 g/kg/day through enteral feeding tubes.

Mai Luo Shu Tong [20–23] granules were purchased from Lu Nan Hou Pu Pharmaceutical Company Limited (approval number Z19991025). The main herbal ingredients were Huangqi* (Astragalus mongholicus)*, Jinyinhua* (Lonicera japonica)*, Huangbo* (Phellodendron chinense)*, Chishao* (Paeonia veitchii)*, Yiyiren* (Coix lacryma-jobi)*, Xuanshen* (Scrophularia ningpoensis)*, Danggui* (Angelica sinensis)*, Baishao* (Cynanchum otophyllum)*, Gancao* (Glycyrrhiza uralensis)*, Shuizhi* (Whitmania pigra)*, Wugong* (Scolopendra subspinipes)*, and Quanxie* (Scorpio)*. It was commonly used in the treatment of superficial thrombophlebitis. They were also administered to patients with nonacute deep vein thrombosis, presenting with lower limb swelling, pain, dull-red coloration, or thread-like vein protrusion. Previous research papers [24] had shown that Mai Luo Shu Tong granules lowered levels of von Willebrand factor (vWF), a unique marker of endothelial damage, which in turn reduced risk of coagulation and thrombosis. In addition, a unique marker associated with platelet activation, cell differentiation 62p (CD62p), was also reduced, further inhibiting blood clot formation. Using a similar dosage conversion, the animal models were given a dose of 2.80 g/kg/day thrice daily.

### 2.2. Experimental Procedure

#### 2.2.1. Induction of Acute Superficial Thrombophlebitis by Mannitol and Experimental Procedure

The acute superficial thrombophlebitis was induced using a 20% mannitol solution (China Resources Double-Crane Pharmaceutical Co., Beijing, China). The dose was 4 ml/kg, twice a day, 6 hours apart, at a rate of 1 ml/min for 7 days. The location of puncture was at the rabbit auricular veins, 2 cm apart from ear tip; during intravenous drip infusion, extravasations of drugs should be avoided. Each infusion was fixed at the puncture site, and all animals were used simultaneously in two ears.

After modeling, the morphological changes of rabbits and the skin color of the tissues around the vein were observed every day. Two samples of the rabbit's ear vein were obtained after the experiment. The specimens of the blank group, the model group, the herbal ointment group, and the positive drug group were collected at the seventh day after the medication. The living specimens in rabbits were collected under phenobarbital intraperitoneal anesthesia. The auricle tissue of 1 cm^2^ was cut at the center of 1.5 cm ahead of the venipuncture site. Part of each rabbit's auricular vein tissues was put into the liquid nitrogen for the PCR, WB detection, and the other part was put into the polyformaldehyde solution, for the production of pathological examination. The evaluation of the result was performed by a single observer who was blinded to the experiment of the specimen.

Rabbit interleukin 6 (IL-6) enzyme-linked immunosorbent assay (ELISA) kit, rabbit interleukin 1*β* (IL-1*β*) ELISA kit, rabbit tumor necrosis factor ELISA kit, and C-reactive protein ELISA kit were obtained from Cusabio Biotech Company Limited.

Horseradish peroxidase (HRP) conjugated anti-mouse immunoglobulin G Super Vision Assay Kit (product code SV0001) and HRP conjugated anti-rabbit immunoglobulin G Super Vision Assay Kit (product code SV0002) were purchased from Boster Biological Technology.

Diaminobenzidine (DAB) HRP Color Development Kit (product code ZLI-9018) was bought from Beijing Golden Bridge Biotechnology Company Limited.

Trizol Total RNA Extraction Kit was obtained from Tiangen Biotech (Beijing) Company Limited.

PrimeScript™ RT Reagent Kit with gDNA Eraser, SYBR® Premix Ex Taq™ II (Tli RNaseH Plus), ROX plus, and DL2000 DNA Marker were acquired from Takara Bio Company.

Primers were purchased from Invitrogen, Thermo Fisher Scientific Corporation.

For the following procedures, the antibodies were prepared as shown in Table 1.

### 2.3. Test Method

#### 2.3.1. Animal Grouping

*Grouping Results.* 6 rabbits were in the blank control group (without any intervention), 6 rabbits in the model group, 6 rabbits in the phlebitis herbal ointment group, and 6 rabbits in the positive drug group (Mai Luo Shu Tong granules). After the model was successfully made, the intragastric administration method of drug was used. Before the administration, the animal medicine groups were fasted for 2 h, and intragastric administration was given twice a day.

#### 2.3.2. Methods of Administration

*Phlebitis Herbal Ointment Group.* 24 h after the model was successfully performed, the drug was administrated intragastric. The administration was given twice a day. The dosage of phlebitis ointment was 2.34 g/kg/d, and the dosage lasted for 7 days.

*Positive Drug Group (Mai Luo Shu Tong Granules).* 24 h after the model was successfully made, intragastric administration method was used. Before the administration, the animals were fasted for 2 h. The intragastric administration was given 3 times a day. The dosage of Mai Luo Shu Tong granules was 2.80 g/kg/d, and the dosage lasted for 7 days.

*Blank Control Group.* No intervention was given.

*Model Group.* No intervention was given after the model was performed.

#### 2.3.3. Methodologies

*(1) ELISA Method.* In order to measure the levels of tumor necrosis factor-*α* (TNF- *α*), interleukin-6 (IL-6), C-reactive protein (CRP), and interleukin-1 (IL-1), ELISA method was employed. According to kit instructions steps, blank holes were added with 100 *μ*L of sample diluent; standard hole and test sample holes were prepared with 100 *μ*L of standard or 100 *μ*L of sample. After sealing the ELISA plate with the adhesive strip, it was incubated at 37°C for 2 hours. The ELISA plate was then air-dried, before 100 *μ*L of biotin antibody was pipetted into each hole and incubated at 37°C for an hour. The plates were washed thrice with the wash buffer provided. Subsequently, 100 *μ*L of HRP-avidin was aliquoted to each well and incubated at 37°C for another hour. The microplate was then rinsed five times with wash buffer to ensure that all impurities were removed. After the microplate was thoroughly washed, 90 *μ*L of TMB substrate was added and incubated in the dark at 37°C for 15 minutes. Then, 50 *μ*L of stop solution was pipetted into each well; the order of adding was the same as the tetramethylbenzidine (TMB) substrate before. The optical density of each well was then determined by using a microplate reader which was set to a wavelength of 450 nm.

*(2) Hematoxylin and Eosin Staining.* Sample embedded in paraffin was manually cut into slices with thickness 4 *μ*m. After baking at 60°C for an hour, the sample was deparaffinized with dimethylbenzene I for 10 minutes and dimethylbenzene II for another 10 minutes. After retrieving the sample, it was washed with 100% ethanol twice for 5 minutes each, 95% ethanol for 5 minutes, 80% ethanol for 5 minutes, and distilled water for 5 minutes. The washed sample was then stained in hematoxylin for 5 minutes before the excess was washed off and differentiated in acid alcohol for 1 second. Washed with distilled water, the sample was stained with eosin for 10 minutes. Following the staining, the sample was washed with 80% ethanol for 1 second, 95% ethanol for 10 seconds, 100% ethanol for 3 minutes, 100% ethanol for 4 minutes, dimethylbenzene II for 5 minutes, and dimethylbenzene I for 5 minutes. After which, the sample was sealed with neutral balsam and observed under microscope.

*(3) Immunostaining Assay.* 48 auricle tissues were collected. Institutional Research Ethics Committee approval was obtained prior to the use of these materials for research purposes.

*(4) Real-Time Polymerase Chain Reaction for NF-КB p65, PKC mRNA Expression.* Evaluation of mRNA expression was performed through real-time polymerase chain reaction as previously described [25]. Total RNA was extracted from the vein tissue samples by using extraction kits (Tiangen Biotech, Beijing, China). After reverse transcription, PCR amplification was performed with the TRIzol method (TRIzol reagent; Invitrogen Life Technologies, Carlsbad, CA, USA). The real-time PCR primer sequences for target genes were as follows:

5′-TACGATGGAACTACACCCCTGC-3′/5′- TGTGAACTCTGGCTCATACGGT-3′ for NF-КB p65, 5′-CGTCCTGCTGTATGAGATGCT-3′/5′- GGACAAGGATTTGGGGTAGG-3′ for PKC, 5′- AAGTGCGACGTGGACATCCG-3′/5′- GGGCGGTGATCTCCTTCTGC-3′ for GAPDH (Tiangen Biotech, Beijing, China).

For real-time PCR, the cycling conditions were 95°C for 30 min and 40 × (95°C for 5 s, 60°C for 40 s), followed by a melting curve analysis-based assay with conditions of 95°C for 10 s, 60°C for 60 s, and increase in temperature to 95°C for 15 s. Relative expression was assessed by calculating the expression relative to that of GAPDH by using the 2^−ΔΔCT^ method.

*(5) Western Blotting for Detection of NF-КB p65, PKC Expression.* Western blot analysis was performed as described previously [26]. Proteins were isolated from ice-cold vein tissues. Protein concentrations were determined by using the bicinchoninic acid (BCA) assay (Cwbiotech, Beijing, China). The proteins were then separated using 10% SDS-PAGE for 1.5 h before being transferred to polyvinylidene fluoride (PVDF) membranes. The membranes were probed with PKC (1 : 1000), NF-КBp65 (1 : 2000), and GAPDH (1 : 1000) antibodies (TDY Biotech, Beijing, China). Each membrane was washed five times for 3 min and incubated with goat polyclonal secondary antibody to rabbit antibodies (S004, TDY Biotech, Beijing, China) and goat polyclonal secondary antibody to mouse antibodies (S001, TDY Biotech, Beijing, China). Finally, densitometry was performed to quantitate protein band intensities by using the Gel Image System ver. 4.00 (Tanon, China).

#### 2.3.4. Statistical Analysis

All data are expressed as mean ± standard deviation (SD) values. SPSS ver. 23.0 software (IBM Corp., Armonk, NY, USA) was used for statistical analyses. The data were compared between groups using one-way analysis of variance (ANOVA), followed by Student's *t*-tests. Two pairs of comparison were used: LSD, Dunnett-*t* test. *P* < 0.05 was considered statistically significant. The results of ELISA test and pathological image analysis were statistically analyzed. The original data can be found at Beijing University of Chinese Medicine (Dr. Guangzong Li).

## 3. Results

### 3.1. General Conditions of Animals

**Figure 1:**
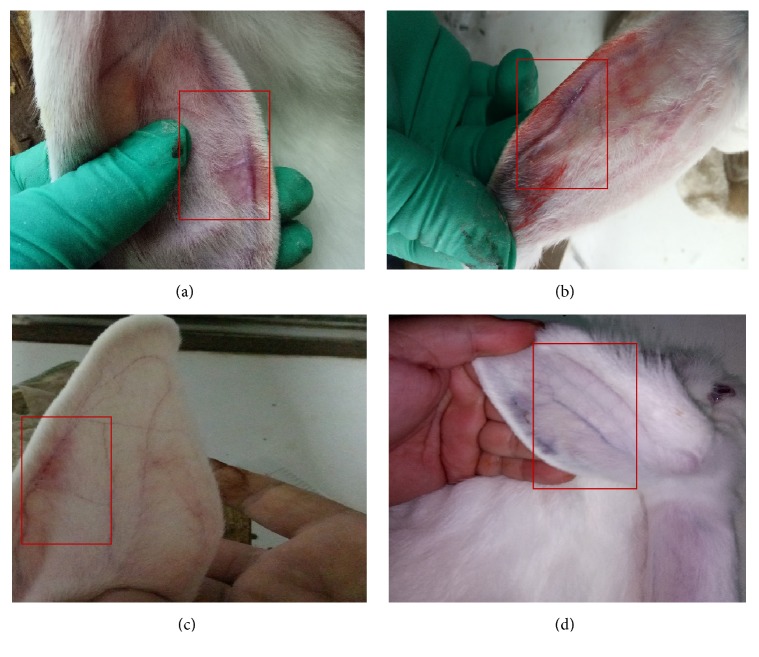
Phlebitis (a–d).

All animals tolerated the entire experiment well, and no deaths occurred. An ear swelling occurred after 3 to 5 days after the rabbit ear margin vein model (Figure 1). Puncture point of peripheral venous was inflamed, swelling, and cord-like, accompanied by yellow urine, activity and food intake decreasing, and fewer water intake. After the administration, the inflammation of the puncture point was reduced in the herbal ointment group and also in the positive drug group. In addition, the activity and diet returned to normal in these both groups. The recovery situation in herbal ointment group was better than that in the positive drug group.

### 3.2. The Influence of Phlebitis Ointment on Inflammatory Factors

Table 2 shows the results of the inflammatory factors in the four different groups.

**Figure 2:**
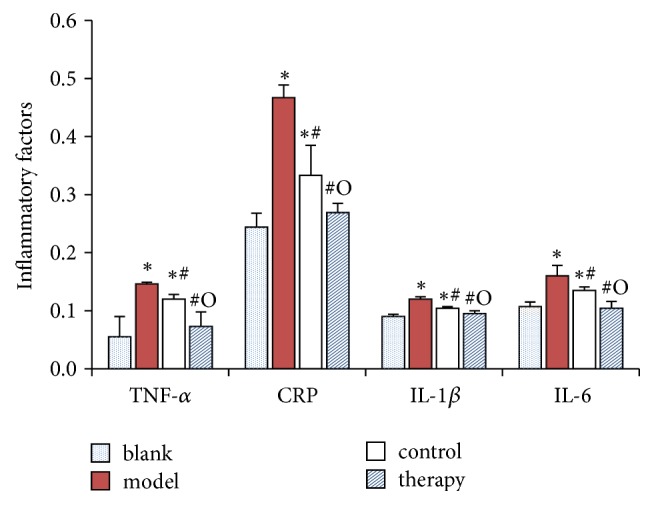
Categorical data of inflammatory factor in the rabbit blood.

Compared with the blank group, the levels of TNF-*α*, CRP, IL-1*β*, and IL-6 in the serum of the model group were significantly increased (*P* < 0.05). Compared with the model group, the levels of TNF-*α*, CRP, IL-1*β*, and IL-6 in serum were decreased in intravenous ointment group and positive drug group. Intravenous ointment could reduce the level of inflammatory factors more than positive drug (Figure 2).

### 3.3. Pathological Results

**Figure 3:**
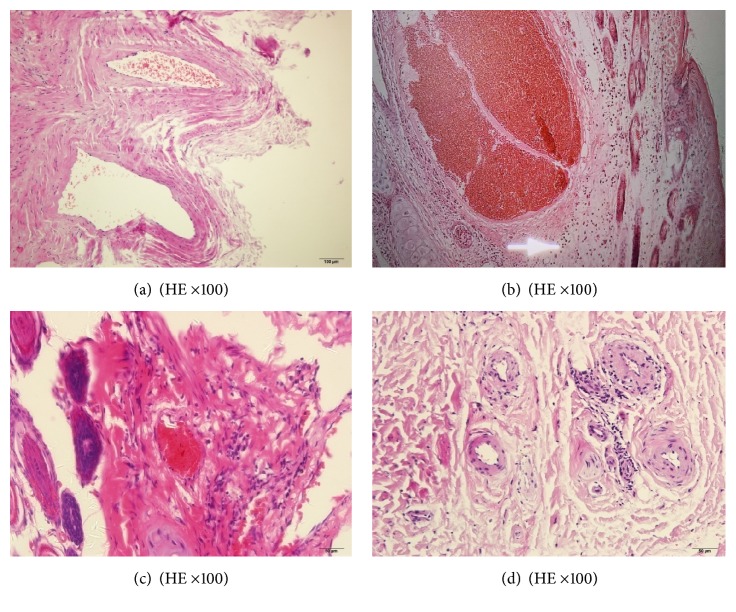
Pathological analyses ((a) blank, (b) model, (c) control, (d) therapy).

Pathological analysis of the blank group showed that the tunica intima was smooth and arranged uniformly and the uniform endothelial cells were spindle shaped and arranged orderly. Similarly, the tunica media displayed spatial order and showed no forms of hyperplasia or dysplasia (Figure 3(a)). In the animal model group, the scaffold of the venous tunica media was loosened, and the tunica intima was thin and inelastic. In addition, the vascular lumen was irregular and showed growth of thrombus. Inflammatory cell infiltration was also observed in the vascular walls (Figure 3(b)). As for the control group with positive drug, a small thrombus was found in the vascular lumen under a light microscope, and the vascular walls showed slight thinning with inflammatory infiltration. Tunica intima displayed no substantial shedding, while the tunica media was loose (Figure 3(c)). In the therapy group with “phlebitis ointment,” vascular structure was clear, obvious thrombus and thinning of blood vessel were not observed, there was no exfoliation of vascular endothelial cells, smooth muscle tissue structure of vascular was neat, some inflammatory cell focal infiltration in the vascular wall and between the tissues could be found, and lymphocytes were visible (Figure 3(d)).

The infiltration of control group and therapy group was less than that of model group. There is some small thrombus filled in the vascular lumen and vascular endothelial cells without damage in the control group.

**Figure 4:**
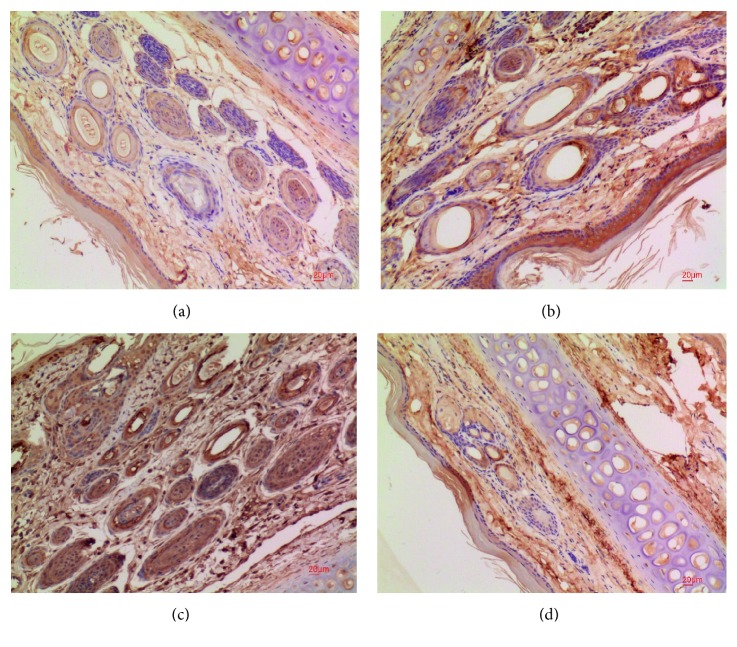
Immunohistochemistry IHC ×100 ((a) therapy, (b) control, (c) model, (d) blank).

Typical results from the immunohistochemistry investigations are shown in Figure 4.

Pathological findings are described in the following paragraph. In the blank group, the intima was smooth in the field of vision, the endothelial cells were spindle shaped, arranged in order, uniform in size, and smooth in the vascular membrane smooth muscle, without proliferation or degeneration (Figure 4(d)). In the model group, the smooth muscle of the vein membrane was loose, the inner membrane was thin, there was no inner elastic membrane, the vein wall was thinner and collapsed, the lumen was irregular, there was thrombus inside, and the infiltration of some inflammatory cells can be seen in the blood vessel wall and between the tissues (Figure 4(c)). In the positive drug group, under the light microscope, some micro thrombi were filled in the vascular lumen, vascular wall became thin, vascular endothelial cells had no obvious exfoliation, the structure of vascular smooth muscle was loose, and the infiltration of some inflammatory cells can be seen in the blood vessel wall and between the tissues (Figure 4(b)). In the phlebitis ointment group, vascular structure was clear, without obvious thrombosis, the vascular wall was not thin, there was no vascular denudation of endothelial cells, the smooth muscle tissue of vessels is of regular structure, some inflammatory cells were observed in the walls and tissues of the vessels, and lymphocytes were seen (Figure 4(a)). The positive drug group was compared with the phlebitis herbal ointment group: In the positive drug group, some micro thrombi appeared in the lumen of the vessel and caused no damage to the vascular endothelial cells. The inflammatory cell infiltration in the two groups was less than that in the model group.

NF-КBp65 protein expression and NF-КBp6 positive expression were mainly concentrated in the cytoplasm of endothelial cells. The expression of NF-КBp65 in the therapy and model group was highly positive. The model group showed slight positive expression at a higher level. The therapy group showed weak positive expression. The blank group was weaker than the therapy group.

### 3.4. Real-Time PCR

The changes of target gene expression in rabbit tissue samples were also detected (Figure 6).

#### 3.4.1. RNA Electrophoresis Chart

**Figure 5:**
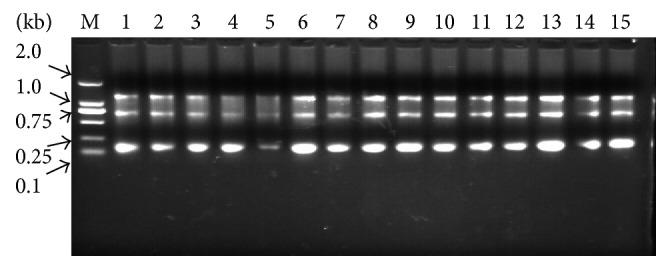
RNA electrophoresis chart (M: DNA Marker: DM2000).

**Figure 6:**
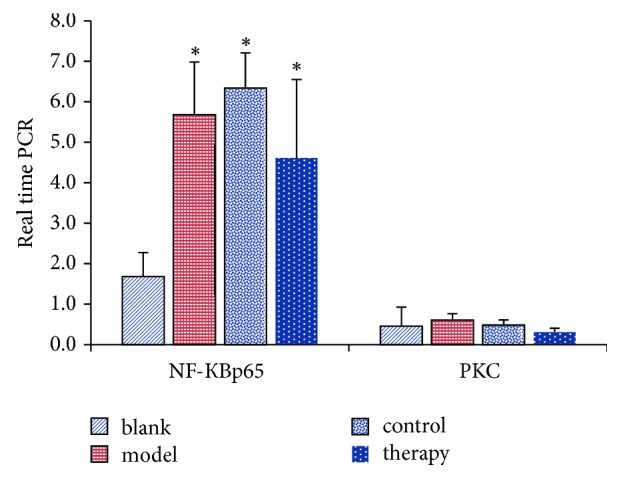
Real-time PCR detection of relative quantitative results.

We take 5 *μ*L of RNA and run on a 1% agarose gel. The result was as shown in Figure 5.

#### 3.4.2. The Relative Quantitative Analysis Results of Each Sample

Volume of each sample during real-time quantitative PCR was 2 *μ*L. However, due to RNA concentration quantitative error and RNA reverse transcription efficiency error, the volume of 2 *μ*L of cDNA per sample was not exactly the same. To correct this difference, we used the internal parameters (the amount of expression between the different samples was essentially constant) as the correction. This was done according to the relative quantitative formula of 2^−ΔΔct^.

We calculated the relative quantitative results of the target gene for each sample. The results are shown in Table 3.

The therapy of NF-КBp65 and PKC in the group was higher than that in the control group. The levels of NF-КBp65 and PKC in the control group were lower than those in the model group (Figure 9).

### 3.5. Western Blot

**Figure 7:**
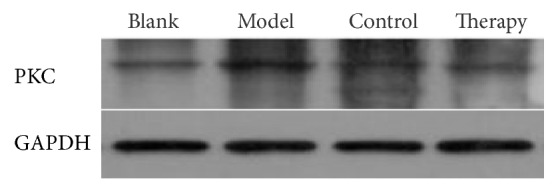
PKC Western blot bands.

**Figure 8:**
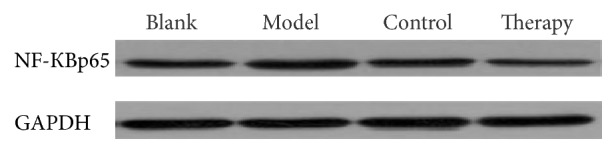
NF-КBp65 Western blot bands.

**Figure 9:**
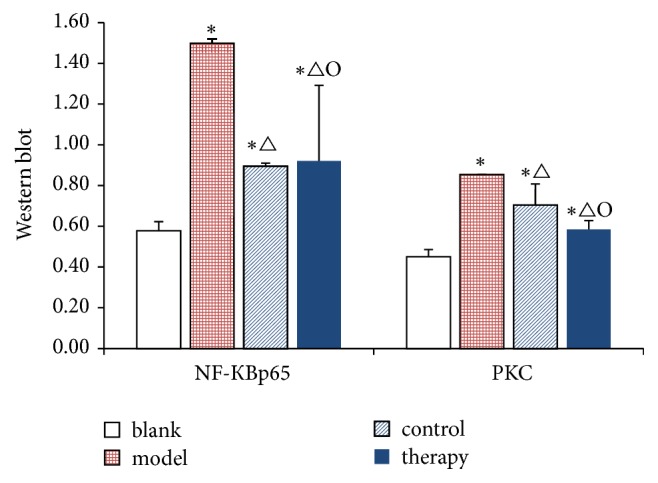
Western blot test results.

The Western blot protocol and results are shown in Figures 7–9 and in Table 4.

The expression of NF-КBp65 protein in blank group rabbits was lower than that in the model group, control group, and therapy group. This is consistent with the results of rabbit plasma protein in the previous section. Compared with the blank group, the expression of NF-КBp65 in the model group, control group, and therapy group was significantly lower (^*∗*^*P* < 0.05). Compared with the model group, the expression of NF-КBp65 in the control group and therapy group was significantly lower and the difference was significant (^Δ^*P* < 0.05). The therapy group compared with the control group NF-КBp65 resulted in *O* < 0.05. The PKC of model group, control group, and therapy group compared with the blank group resulted in ^*∗*^*P* < 0.05. Compared with the model group, the PKC of the control group and the therapy group was significantly decreased (^Δ^*P* < 0.05). The PKC of the therapy group compared with that of the control group was *O* < 0.05.

## 4. Discussion

The results obtained using good experimental designs are well documented. At the same time, the results are important for general acceptance of the traditional Chinese medical treatment in the world. A transfusion thrombophlebitis model of Japanese rabbits was established successfully by mannitol intravenous injection. Through animal experiments, it was found that after five to seven days of modeling, the auricular veins were red and swollen one after another. When touched, it felt like having a cord (Figure 1), and there was a decrease in activity, appetite, water, and so on. After the administration, the symptoms of the rabbits in the phlebitis herbal ointment group were better than those in the positive drug group, and the improvement of symptoms in the two groups was better than that in the model group.

Due to the content detection of serum tumor necrosis factor alpha (TNF-*α*), interleukin 6 (IL-L6), C-reactive protein (CRP), and IL-1 beta through enzyme-linked immunosorbent assay, it was found that phlebitis herbal ointment can reduce the levels of tumor necrosis factor alpha (TNF-*α*), interleukin 6 (IL-L6), C-reactive protein (CRP), and IL-1 *β*. At the same time, the protein quantitative detection confirmed that the expression levels of NF-КBp65 and PKC gene in the herbal ointment group and positive drug group were significantly changed. The changes of NF-КBp65 and PKC gene expression level may be an important factor in the cure of thrombophlebitis.

Concerning the effects of inflammatory markers on acute superficial thrombophlebitis, Roumen-Klappe et al. and other authors [27–30] found that the expression of TNF-*α*, IL-6, CRP, and other inflammatory markers in vivo plasma increased in patients with venous thrombosis.

Tumor necrosis factor alpha (TNF-*α*) is an important mediator of inflammatory response and may cause the release of other inflammatory factors. In the acute stage of inflammatory reaction, plasma TNF-*α* can regulate the process of vascular inflammation and affect the activity of vascular endothelial cell [7].

Interleukin 6 (IL-6), is an important factor in inflammatory response. It has a variety of biological activities and plays an important role in immune regulation and stress response. It can stimulate the production of plasminogen activator inhibitors in the liver, and it binds with plasminogen activator and makes them lack activity, leading to the decrease of fibrinolysis and thrombosis. In this experiment, IL-6 in the model group was significantly increased than that in the positive drug group, herbal ointment group, and blank group, which indicates that IL-6 increases obviously in the acute stage of superficial thrombophlebitis. After treatment, the IL-6 value in the positive drug group and the herbal ointment group decreased compared with the model group, which demonstrates that phlebitis herbal ointment and Mai Luo Shu Tong granules can reduce the IL-6 level in Japanese rabbits, but the level of IL-6 in phlebitis herbal ointment group was shown in this study to be lower than that in the positive drug group.

C-reactive protein (CRP) is the first acute phase protein associated with inflammation and is a relatively sensitive indicator of inflammatory response [31]. When inflammation occurs, it stimulates the liver to synthesize CRP and activates the complement through classical pathways, which can release inflammatory mediators and promote adhesion and phagocytosis [32]. It can also induce monocytes to express procoagulant factor-tissue factor (TF), which leads to thrombosis. In our experiment, CRP in the model group was significantly increased than that in the positive drug group, herbal ointment group, and blank group. This reveals that CRP increased significantly in the acute stage of thrombophlebitis. After treatment, the IL-6 value in the positive drug group and the herbal ointment group decreased compared with the model group, but it was still higher than that in the blank group.

IL-1 *β* is produced by activated monocyte macrophage cells [33], mainly involved in inflammatory responses. It promotes leukocyte migration and binding to endothelial cell IL-1 *β* receptors. IL-1 *β* activates transcription of target genes through the NF- *κ* B signalling pathway, activates prothrombin, and promotes thrombosis. The release of a large number of IL-1 *β* proteins results in the accumulation of activated neutrophils on the vessel wall, which increases the activity of the blood coagulation and leads to the formation of thrombus [34].

In animal experiments, we found that the levels of TNF- *α*, IL-L6, CRP, and IL-1*β* in plasma of model group were significantly higher than those in the blank group. After drug treatment, the levels of TNF- *α*, IL-L6, CRP, and IL-1*β* in the positive group and herbal ointment group decreased significantly, compared with the model group. Compared with the positive drug group, the levels of TNF- *α*, IL-L6, CRP, and IL-1*β* in the herbal ointment group decreased significantly. The local inflammatory reaction can destroy the tissue and reduce the excessive synthesis and release of TNF-*α*, IL-L6, CRP, IL-1 *β*, and other inflammatory mediators, which is beneficial to control the degree of inflammatory reaction and also beneficial to the treatment of acute superficial thrombophlebitis. Local inflammatory reaction can destroy the tissue and reduce the excessive release of inflammatory mediators such as IL-1*β*, TNF-*α*, IL-6, and CRP, which is beneficial to control the degree of inflammatory reaction and then to inhibit the formation of thrombosis. The results of this experiment showed that the levels of IL-1*β*, TNF-*α*, IL-6, and CRP in the serum of rabbits could be reduced by pharmacological intervention. In the Chinese medicine therapy group, the effect of reducing the inflammation was remarkable. Therefore, we consider that the phlebitis ointment can improve the inflammation state of rabbits, which may be related to the treatment of thrombophlebitis.

Zhang et al. [35] found that the inflammatory factors TNF-*α*, IL- l *β*, and IL- 6 had different expression levels in different thrombus status and different phase points. Process of thrombosis was accompanied by inflammatory reaction.

After consulting the related literature about animal vein thrombosis, we found that, in the course of venous thrombosis, PMN is the major inflammatory cell and the first inflammatory cell to reach the damaged tissue [36]. The previous research data showed that NF- КB can promote the expression of proinflammatory cytokines and profibrotic factors and then aggravate the severity of phlebitis [37]. NF- КB signalling pathway is a signal transduction pathway to inhibit PMN apoptosis. Activation of NF-КB can induce delayed neutrophil apoptosis, prolong its life cycle, and activate and produce a large number of inflammatory mediators and oxygen free radicals [38]. Ward et al. [39] believed that inhibiting the activation of NF- КB can inhibit neutrophil aggregation and activation effectively, promote apoptosis of PMN, and reduce the inflammatory response during thrombosis, thereby blocking thrombosis. The real-time PCR detection found that the level of NF-КBp65 gene expression changed in the herbal ointment group; the level of NF-КBp65 and PKC gene expression also changed obviously in the positive drug group. The Western blot detection found that the expression of NF-КB protein in the blank group was lower than that in the model group, positive drug group, and herbal ointment group, which was consistent with the results of plasma proteins in the previous sections. The difference in NF-КB of the model group, positive drug group, and the herbal ointment group, compared with the control group, was significant. Compared with the model group, the NF-КB in the rabbit tissue of the positive drug group and the herbal ointment group improved significantly. The difference in the NF-КB between the herbal ointment group and the positive drug group was significant. PKC in the model group, positive drug group, and the herbal ointment group, compared with the control group, the difference was significant. Compared with the model group, the PKC in the rabbit tissue of the positive drug group and the herbal ointment group improved significantly. The difference in the PKC between the herbal ointment group and the positive drug group was significant.

The continuous high activity of NF-КB leads to cytokine expression, leukocyte invasion, and inflammation. As a serine threonine protein kinase, PKC participates in the process of cell signalling during the proliferation of vascular smooth muscle cells. Through the phosphorylation of transcription factors, PKC is widely distributed in various tissues. It is involved in cell proliferation, differentiation, expression, and so on. PKC is also involved in regulation of cell cycle and plays a regulatory role in cell growth and apoptosis. Our results have confirmed this effect. IL-1 *β* combined with the IL-1 *β* receptor of the endothelial cell activates the signal pathway. As a result, tissue factor is released, thereby activating the coagulation pathway, activating thrombin in the plasma, promoting fibrinogen to become fibrin, and ultimately promoting thrombosis. In superficial thrombophlebitis, transcription factor NF-КB was activated to promote apoptosis. Tumor necrosis factor alpha (TNF-*α*) is a proinflammatory cytokine produced by macrophages and monocytes, and it is involved in inflammatory and immune responses with IL-1 *β*, IL-L6, and CRP. Through this experimental study, we confirmed the pathogenesis of NF-КB, PKC, and CRP signalling pathway. TNF- *α*, IL-1 *β*, IL- 6, and other inflammatory factors play the pathogenic role in this disease.

*Limitations of This Study.* Due to the limited experimental conditions, the sample size of Japanese white rabbits with large ears is relatively small, and the experimental results need to be confirmed by a large number of samples. At the same time, the time point of blood sampling can be further refined to precise time and state of blood coagulation. Moreover, the effect of the concentration of drugs and their effects on hypercoagulability and inflammation should be further studied.

## 5. Conclusion

In conclusion it can be stated that the phlebitis ointment reduced the levels of necrosis factor-*α*, interleukin-6, C-reactive protein, and interleukin-1ß. At the same time, phlebitis ointment can upregulate the NF-КBp65 and PKC genes in acute superficial thrombophlebitis.

## Figures and Tables

**Table 1:** Antibodies.

Antibody	Company	Product code	Animal origin	Dilution factor
PKC	Abcam	Ab32122	Rabbit	1 : 200
NF-КBp65	RUIYING	RLT5339	Rabbit	1 : 200
Ki67	RUIYING	RLM3064	Mouse	1 : 150

**Table 2:** Inflammatory factors $\left(n=6,\overset{-}{X}\pm s\right)$.

Group	TNF-*α*	CRP	IL-1*β*	IL-6
Blank	0.055 ± 0.035	0.244 ± 0.024	0.090 ± 0.004	0.107 ± 0.008
Model	0.146 ± 0.003^*∗*^	0.467 ± 0.022^*∗*^	0.120 ± 0.004^*∗*^	0.161 ± 0.018^*∗*^
Control	0.120 ± 0.008^*∗*#^	0.333 ± 0.052^*∗*#^	0.104 ± 0.003^*∗*#^	0.135 ± 0.006^*∗*#^
Therapy	0.073 ± 0.025^#○^	0.269 ± 0.016^#○^	0.095 ± 0.005^#○^	0.104 ± 0.012^#○^

Blank: blank control group; model: model group; control: Mai Luo Shu Tong group; therapy: “phlebitis ointment” group. Data are presented as the mean ± SD. ^*∗*^*P* < 0.05 versus the blank control group; ^#^*P* < 0.05 versus the model group; ^○^*P* < 0.05 versus the control group (*n* = 6 per group).

**Table 3:** Real time PCR detection of relative quantitative results (C1 samples as control samples).

Group	N	NF-КBp65	PKC
Blank	6	1.684 ± 0.592	0.454 ± 0.473
Model	6	5.687 ± 1.299^*∗*^	0.603 ± 0.163
Control	6	6.343 ± 0.868^*∗*^	0.484 ± 0.127
Therapy	6	4.598 ± 1.955^*∗*^	0.310 ± 0.094

^*∗*^Compared with the blank group, *P* < 0.05.

**Table 4:** Western blot test results.

Groups	NF-КBp65	PKC
Blank	0.578 ± 0.045	0.451 ± 0.035
Model	1.499 ± 0.022^*∗*^	0.854 ± 0.004^*∗*^
Control	0.895 ± 0.152^*∗*△^	0.704 ± 0.104^*∗*△^
Therapy	0.921 ± 0.371^*∗*△O^	0.585 ± 0.043^*∗*△O^

Blank: blank control group; model: model group; control: Mai Luo Shu Tong group; therapy: “phlebitis ointment group. Data are presented as the mean ± SD. ^*∗*^*P* < 0.05 versus blank control group; ^△^*P* < 0.05 versus the model group; ^O^*P* < 0.05 versus the control group.
